# A rare case of gastroduodenal junction Kaposiform hemangioendothelioma in a cat: pathological findings and follow-up

**DOI:** 10.1007/s11259-026-11195-3

**Published:** 2026-04-07

**Authors:** Cristina-Diana Borfălău, Andrada Negoescu, Raluca Marica, Dragoș Hodor, Andra Bărbulescu, Alice Radulescu, Elena Teodora Vulcan, Claudiu Gal, Irina Amorim, Marian Taulescu

**Affiliations:** 1 Department of Veterinary Pathology, Faculty of Veterinary Medicine, University of Agricultural Sciences and Veterinary Medicine, Cluj-Napoca, Romania; 2 Department of Veterinary Pathology, Synevovet, Bucharest, Romania; 3 A&A Medical Vet Investigații Veterinare, Bucharest, Romania; 4 Department of Internal Medicine, Faculty of Veterinary Medicine, University of Agronomic Sciences and Veterinary Medicine, Bucharest, Romania; 5 Department of Pathology and Molecular Immunology of the Institute of Biomedical Sciences Abel Salazar (ICBAS), University of Porto, Porto, Portugal; 6 Institute of Molecular Pathology and Immunology, University of Porto (IPATIMUP), Porto, Portugal

**Keywords:** Kaposiform hemangioendothelioma, Gastroduodenal, Immunohistochemistry, ERG, Caveolin-1, Feline

## Abstract

Kaposiform hemangioendothelioma (KHE) is a rare vascular neoplasm predominantly affecting pediatric human patients and has rarely been reported in veterinary medicine. We described the clinicopathological features of an uncommon gastroduodenal junction KHE in a 2-year-old male cat presented with a history of intermittent vomiting, acutely worsening over 48 h. Imaging and endoscopic evaluation revealed complete pyloric obstruction caused by an infiltrative mass with enlarged regional lymph nodes, which were surgically excised and examined histologically and immunohistochemically. Specimens were routinely processed and stained using Hematoxylin and Eosin and Masson’s Trichrome. Immunohistochemical analysis was made using an extended panel of endothelial markers, including Erythroblastosis Transformation-Specific Regulated Gene (ERG) and caveolin-1 (Cav-1), which, to the authors’ knowledge, have not previously been applied in feline vascular tumors, in addition to von Willebrand Factor (vWF) and α-smooth muscle actin (α-SMA). Histologically, the gastroduodenal junction exhibited transmural infiltration by a poorly delimited mass composed of spindle cells organized in short fascicles with slit-like vascular lumina, and a swirling growth pattern conferring a glomeruloid morphology. Neoplastic endothelial cells demonstrated immunoreactivity for von Willebrand factor, ERG and Cav-1 and were surrounded by α-SMA positive cells, supporting the diagnosis of KHE. Gastric lymph nodes displayed marked sclerosis and lymphoid atrophy. The cat showed no recurrence or metastasis one year after surgery. To our knowledge, this is the first reported case of a gastroduodenal KHE in animals. This report highlights the value of expanded immunohistochemical panels in differentiating KHE from other vascular lesions and suggests a possible paraneoplastic fibrotic response within regional lymph nodes.

## Background

Vascular anomalies constitute a well-characterized group of disorders in human medicine and are broadly classified into vascular tumors and vascular malformations, each exhibiting distinct biological behavior and prognostic significance (Sbaraglia et al. 2020; ISSVA 2025). The most common vascular tumors in humans are hemangiomas, affecting mostly infants, and angiosarcomas, especially in the adult population (Portugal et al. 2023; Richter and Friedman 2012). In veterinary medicine, vascular tumors are common in dogs, occasionally observed in cats, but rare in other domestic or wild animals (Miller et al. 1992). Across animal species, the most frequent vascular tumor diagnosed is represented by hemangiosarcoma (Griffin et al. 2021). Other vascular tumors include hemangiomas and hemangioendotheliomas, the latter of which are histologically classified into epithelioid, retiform, and kaposiform (Roccabianca et al. 2020).

Kaposiform hemangioendothelioma (KHE) is a rare vascular tumor, characterized as a borderline neoplasm due to its locally aggressive behavior. It is most commonly diagnosed in infants and children, whereas occurrence in adults is uncommon (Ji et al. 2020). In veterinary medicine, this lesion is classified as an intermediate vascular tumor and is rare among animal species, with a low number of documented cases in dogs, cattle, and one bird (Vincek et al. 2004; Pires et al. 2009; Paździor-Czapula et al. 2015; Rossi et al. 2016; Epikmen et al. 2020). In animals, KHE was found in the heart (Paździor‐Czapula et al. 2015), skin (Vincek et al. 2004) and urinary bladder (Pires et al. 2009). In cats, KHE has previously been described in the external genital mucosa of a male cat, as reported in a conference communication (Ateş and Bulut 2022).

Diagnosis of KHE is based on characteristic histopathological features and immunohistochemical analysis for endothelial origin confirmation, using specific endothelial cell markers, including CD31, CD34, Factor VIII–related antigen (vWF) and Erythroblastosis Transformation-Specific Regulated Gene (Liu et al. 2015).

The Erythroblastosis Transformation-Specific Regulated Gene (ERG) is a crucial member of the E-26 transformation-specific (ETS) family of transcription factors, recognized for its pivotal role in endothelial cell differentiation and angiogenesis (Birdsey et al. 2011), showing high sensitivity and specificity for these cells, making it a valuable marker for distinguishing vascular tumors from other spindle cell or poorly differentiated neoplasms. In addition, caveolin-1 (Cav-1) is a structural protein and a principal component of caveolae, small invagination of the plasma membrane, that are involved in numerous cellular processes, including signal transduction, cholesterol homeostasis, endocytosis, cell proliferation and differentiation, and apoptosis (Dalton et al. 2023). Caveolin-1 may be used as a diagnostic marker for vascular tumors, particularly for distinguishing benign from malignant forms (Morgan et al. 2001).

A search of Google Scholar, PubMed, CAB Direct, Web of Science, and Scopus using the terms “Kaposi”, “Kaposiform”, “Hemangioendothelioma”, “sarcoma”, “animals”, “cat”, and “feline”, yielded no records of feline gastroduodenal Kaposiform hemangioendothelioma, suggesting that this condition has not been previously reported in cats.

This study aims to provide a comprehensive description of the pathological features and clinical outcome of this rare vascular tumor, and to contribute to its characterization through the use of a novel immunohistochemical panel, including ERG and Cav-1, markers that have not previously been investigated in veterinary pathology. This is complemented by a comprehensive review of the existing literature on this neoplasm in animals and its principal differential diagnoses.

## Case presentation

A 2.6-year-old neutered male Persian cat (3 kg) was presented as an emergency with acute vomiting, abdominal pain and distention worsening over 48 h. The cat was strictly indoor, fed a commercially balanced diet, vaccinated until one year of age, and had not received regular deworming.

On presentation, the cat was dull and weakly responsive, with marked abdominal distention and cranial abdominal pain. Moderate dehydration was noted, along with subnormal body temperature (36 °C), pale mucous membranes, and normal capillary refill time (< 2 s). Respiratory rate was mildly elevated (38 breaths/min; reference interval: 20–30), but thoracic auscultation was unremarkable. Cardiac auscultation revealed a mild left-sided systolic murmur (grade II/VI), heart rate of 180 bpm (reference interval: 120–140), and normal femoral pulses. Systolic blood pressure, measured via Doppler on the dorsal pedal artery, was 170 mmHg (reference interval:100–140). Complete blood analysis revealed leukocytosis (23.5 × 10⁹/L; reference interval: 5.5–19.5) with neutrophilia (19.75 × 10⁹/L; reference interval: 2.32–12.58). The cat also showed severe azotemia (BUN: 113.4 mg/dL; reference interval: 17.6–32.8, Creatinine: 3.21 mg/dL; reference interval: 0.8–1.8), hyperproteinemia (8.1 g/dL; reference interval: 5.7–7.8) with hyperalbuminemia (4.6 g/dL; reference interval: 2.3–3.5), elevated AST (77 U/L; reference interval: 18–51), hyperglycemia (245 mg/dL; reference interval: 71–148), and hypercholesterolemia (201 mg/dL; reference interval: 89–176). Electrolyte imbalances included severe hyponatremia (115 mEq/L; reference interval: 147–156), hypokalemia (1.9 mEq/L; reference interval: 3.4–4.6), and hypochloremia (53 mEq/L; reference interval: 107–120). These findings suggested marked dehydration, a prerenal component, and metabolic dysfunction contributing to the clinical signs.

**Fig. 1:**
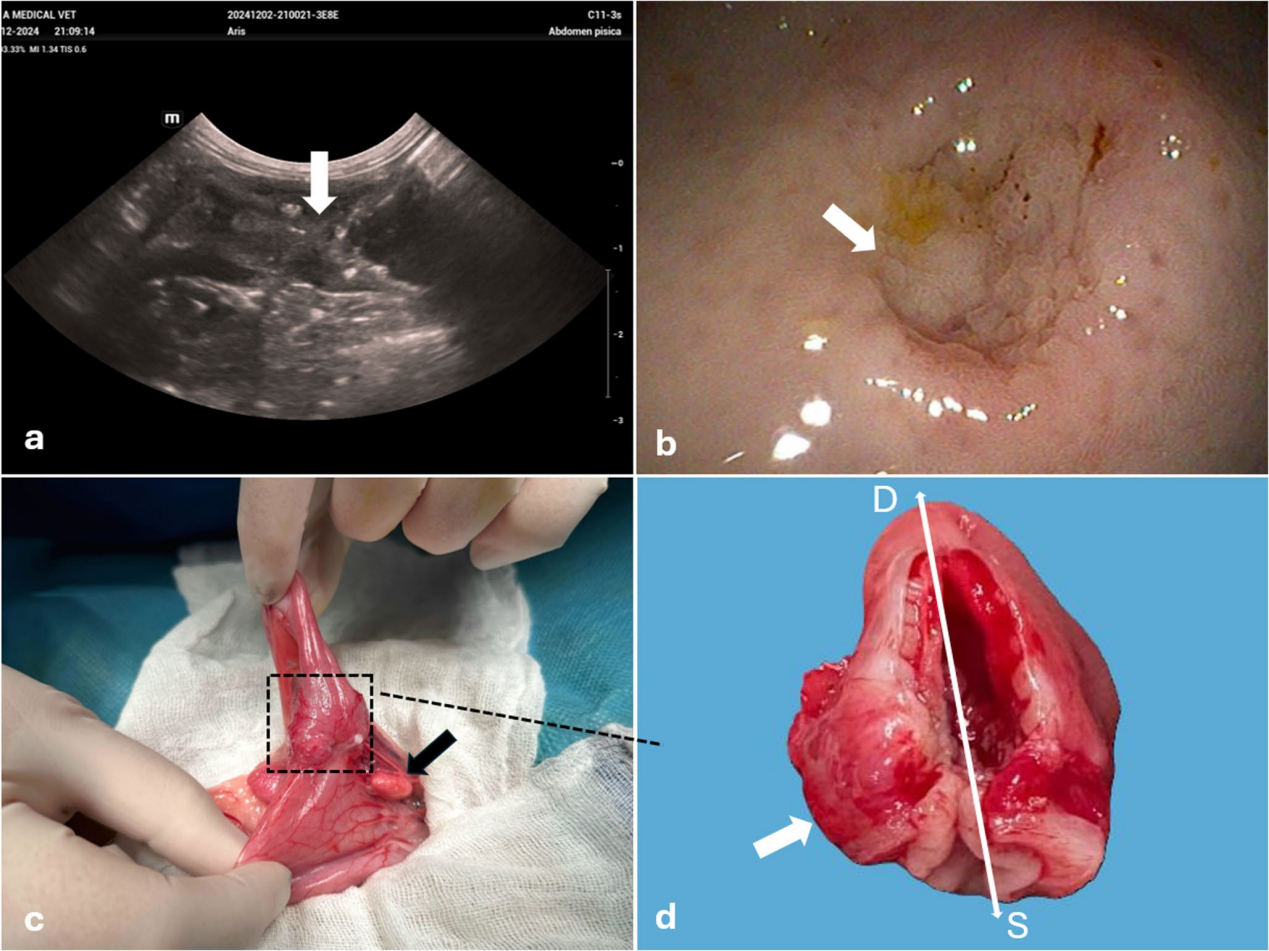
Imaging and gross features of the feline gastroduodenal junction KHE (**a**) Abdominal ultrasonography of the pylorus showing marked circumferential wall thickening (arrow), heterogeneous echogenicity, and loss of normal layering, resulting in complete luminal obliteration. **b**) Endoscopic view of a severe intraluminal proliferative lesion causing complete pyloric occlusion with no duodenal passage (arrow). **c)** Intraoperative view of a markedly thickened, firm gastroduodenal junction wall (delimited area) and an enlarged gastric lymph node (arrow). **d**) Gross appearance of the resected tissue showing severe wall thickening (arrow) with near-complete to complete luminal occlusion; S (stomach/ gastric region), D (duodenal region)

Radiographs (left lateral and ventrodorsal views) showed severe gastric dilation with gas and fluid, along with gaseous intestinal distension, suggesting gastric outflow obstruction, severe functional ileus, or a condition resembling gastric dilatation-volvulus (GDV), although rare in cats. Abdominal ultrasound revealed a stomach markedly distended with fluid and gas, occupying the entire abdominal cavity. At the pylorus, wall layering was lost, the wall was thickened (6.3 mm), and the luminal surface appeared hypoechoic and irregular with hyperechoic intraluminal inclusions (Fig. 1a). A reactive lymph node adjacent to the pylorus measured 7.8 × 6.4 mm. The jejunum, displayed caudally, showed mild wall thickening (3.5 mm) with a muscularis-to-mucosa ratio of 1:1, suggesting a possible chronic inflammatory pattern, without pre-stenotic dilation. No free peritoneal fluid was observed. Additional findings included bilateral polycystic kidney disease, hepatopathy (starry-sky liver), pancreatitis, and subacute or mild biliary stasis. The pyloric wall changes and gastric distention were consistent with gastric outflow obstruction, possibly due to severe inflammation, chronic ulcer, or an infiltrative neoplasm. Enlargement of the adjacent gastric lymph node supported an inflammatory or neoplastic process.

During esophagogastroduodenoscopy, the esophagus appeared structurally normal but showed marked mucosal hyperemia, suggesting reflux-related irritation. The stomach was severely distended, containing hair, liquid, and partially digested food. An infiltrative lesion at the pylorus caused complete luminal obstruction (Fig. 1b), consistent with prior ultrasound findings of wall thickening and loss of normal layering. Due to the lesion’s extent and obstructive nature, conversion to open exploratory surgery was recommended to allow resection of the affected pyloric region and assessment of additional intra-abdominal pathology not visible endoscopically.

The patient was classified as ASA III due to systemic compromise, including azotemia, electrolyte imbalances, and cardiovascular concerns. A balanced, multimodal anesthetic protocol was used to minimize hemodynamic depression and allow intraoperative adjustments. Induction was achieved with co-induction using midazolam (0.1 mg/kg IV), ketamine (0.5 mg/kg IV), and propofol (1 mg/kg IV) titrated to effect, followed by intubation with a 3.5 mm flexometallic endotracheal tube connected to a rebreathing system. Anesthesia was maintained with isoflurane in oxygen, adjusted as needed, with Ringer’s Lactate administered as a continuous IV infusion based on blood pressure and perfusion. Intraoperative analgesia included a fentanyl bolus (2 µg/kg IV) for heart rates exceeding 160 bpm, and dobutamine (2 µg/kg/min) was infused for inotropic support. Antibiotics consisted of cefotaxime (100 mg/kg IV) intraoperatively and metronidazole (15 mg/kg IV over 20 min) at the end of surgery. A midline incision was made from the subxiphoid region to midway between the umbilicus and pubis.

Exploratory laparotomy revealed that the pyloric region was markedly dense, gray-red, irregular and thickened, with numerous adhesions (Fig. 1c), resulting in complete luminal occlusion. Both cranial and caudal gastric lymph nodes were enlarged and reactive. The affected pyloric segment and adjacent antral stomach were resected, and a gastrojejunostomy was performed to restore gastrointestinal continuity. Resected tissues were submitted for histopathological evaluation (Fig. 1d). The abdominal cavity was lavaged with sterile saline, and a Jackson-Pratt (JP) drain was placed and secured with cruciate dermal sutures. Postoperative analgesia included buprenorphine (0.02 mg/kg IV every 8 h). Intraoperative complications included hypothermia (32.3 °C), hypotension (80 mmHg), and mild bradycardia (80 bpm) during early anesthesia. Recovery was slow due to hypothermia, with gradual improvement following active warming.

Tissue samples were fixed in 10% neutral buffered formalin. After paraffin embedding, sections of 2 μm were obtained and stained with hematoxylin and eosin (H&E), and Masson Trichrome (MT). For immunohistochemical analysis, the following antibodies were used: anti-CD31 (ready to use, clone JC70A, Cat. no. PA0414, Leica Biosystems), anti-von Willebrand Factor (vWF) (ready to use, clone 36B11, Cat. no. PA0055, Leica Biosystems), anti-Erythroblastosis Transformation-Specific Regulated Gene (ERG, ready to use, clone: EPR3864, Cat. no. 06478450001, Ventana Medical Systems, Tucson, Arizona), anti-alpha smooth muscle actin (α-SMA, dilution 1:100, polyclonal, Cat. no. ab5694, Abcam) and anti-caveolin-1 (dilution 1:300, polyclonal, Cat. no. 610059, BD Biosciences). The technique was performed with an automated immunostainer for ERG-antibody (Ventana Benchmark ULTRA, Ventana Medical Systems, Tucson, AZ, USA), with all the reagents being dispensed automatically. For α-SMA and caveolin-1 antibodies, manual processing was conducted with serial dilutions applied after deparaffinization and rehydration. The procedure was performed using a Dako EnVision Flex + kit (mouse, high pH) according to the manufacturer’s instructions, and antigen retrieval was achieved through heat-induced epitope retrieval using Novocastra™ Epitope Retrieval Solution pH9 (1:10 dilution) for 30 min. For CD31, multiple antigen retrieval conditions and protocol variation were tested, including adjustment in buffer composition, pH, and heat-mediated retrieval parameters, in accordance with available veterinary literature. The procedure was repeated on multiple occasions in our laboratory and independently in three additional laboratories (University of Medicine and Pharmacy Cluj-Napoca, University of Porto, Synevovet Laboratories Bucharest), to exclude technical variability. Both internal and external controls were tested. Internal controls consisted of endothelial cells of normal duodenal submucosa blood vessels for CD31, vWF, ERG and Cav-1, and muscular cells from the gastric muscular layers for α-SMA. Furthermore, a case of feline subcutaneous hemangiosarcoma was used as external control for CD31 and vWF. No CD31immunoreactivity was detected in the feline tissues under the tested conditions. A part of the slides was examined with the optical microscope Olympus BX51, and the rest of the slide images were acquired using a digital scanner (IntelliSite Ultra-Fast Scanner, Philips, Best, The Netherlands) at a magnification of 40x. Photomicrographs were captured using an Olympus SP350 digital camera and Stream Basic imaging software, version 1.5.1 (Olympus Corporation, Tokyo, Japan), as well as the Philips Image Management System (software version 3.3.7).

To support the suitability of human-directed antibodies for the expression of ERG and Caveolin-1 in feline normal and tumor tissues, *in silico* amino acid sequence homology analyses were performed. Additionally, we have compared with PECAM (CD31) and von Willebrand factor to further sustain our supposition that ERG it is a viable immunomarker for endothelial cells when it involves cats. Human and feline protein sequences were retrieved from the National Library of Medicine database (National Center for Biotechnology Information – NCBI n.d). Overall sequence similarity between human and feline proteins was first assessed using NCBI BLAST (n.d). Multiple sequence alignments were subsequently conducted using the Clustal Omega algorithm to evaluate conservation across the full-length proteins, with particular attention to regions corresponding to antibody epitopes (Madeira et al. 2024; EMBL-EBI 2026 n.d).

For ERG, which is recognized as a C-terminal epitope (Park et al. 2010), the human sequence (Accession number: NP_891548) and feline sequence (Accession number: XP_011284075) were compared. BLAST analyses demonstrated 92% overall amino acid identity, while alignment of the epitope-containing region (amino acids 393–479) showed 98.9% similarities, with only a single non-identical residue.

A similar approach was subsequently applied to caveolin-1, PECAM-1 (CD31), and von Willebrand factor. Amino acid sequence alignments were performed for human caveolin-1 (Accession number: AAD23745), feline caveolin-1 (Accession number: NP_001019334), human PECAM-1 (CD31) (Accession number: XP_054172404), feline PECAM-1 (CD31) (Accession number: XP_044900077), human von Willebrand factor precursor (Accession number: P04275), and feline von Willebrand factor precursor (Accession number: NP_001233208). BLAST analyses revealed 96% overall identity for caveolin-1, 70% for PECAM-1, and 87% for von Willebrand between human and feline proteins. Evaluation of the reported immunogen regions showed for the first 96.9% (positions 1–97), 79.2% for the second (positions 28–200), and 88.1% (positions 1277–1453, 1691–1871, 2255–2812) for the latter.

**Fig. 2:**
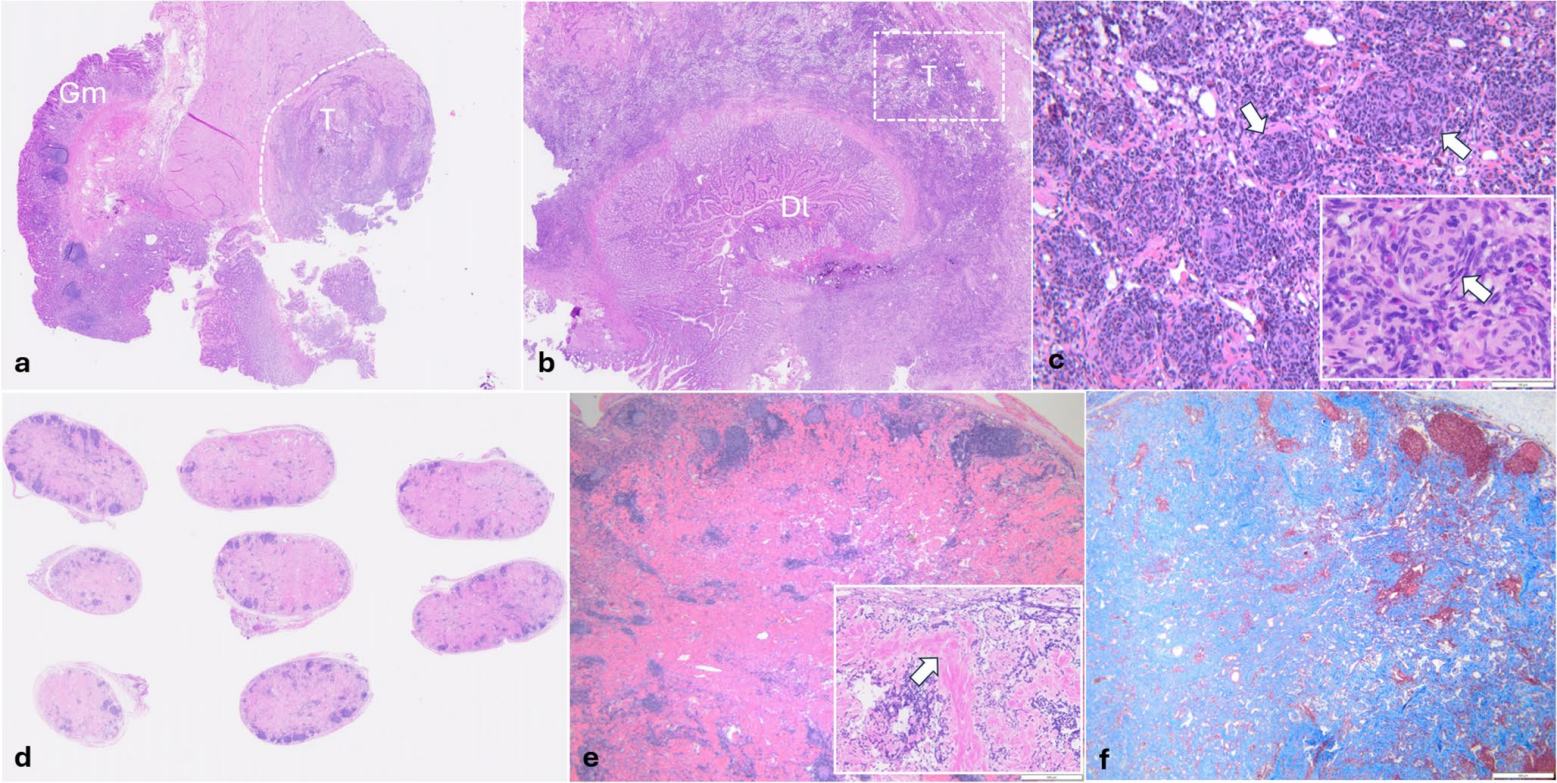
Histological features of the feline gastroduodenal junction KHE (**a**, **b**) The gastroduodenal wall is transmurally infiltrated and replaced by a neoplastic mass (T), Gm (gastric mucosa), Dl (duodenal lumen), H&E stain. **c)** The tumor is composed of spindle to polygonal cells arranged concentrically around variably sized vascular channels, forming slit-like spaces filled with erythrocytes and a glomeruloid pattern (inset, arrows), H&E stain. **d**, **e)** Photomicrographs of the gastric lymph nodes (H&E stain) showing marked sclerosis (inset, arrow) and lymphoid atrophy, confirmed by MT stain (**f**)

Microscopically, the submucosa of the resected gastroduodenal junction was severely distended and infiltrated by poorly demarcated, non-encapsulated, intensely cellular neoplasm, extended into the tunica muscularis (Fig. 2a, b). The neoplastic cells were arranged concentrically around variably sized vascular structures, multifocally forming slit-like spaces filled with erythrocytes and a glomeruloid pattern (Fig. 2c). The cells were spindle to polygonal, with variably distinct borders and small amount of pale, eosinophilic granular cytoplasm. The nuclei were round to oval, occasionally indented, with vesicular chromatin and 1–2 small basophilic nucleoli. Anisocytosis and anisokaryosis were moderate and the mitotic count was 2/10HPF (2.37mm^2^). The fibrovascular stroma was composed of collagen fibers, multifocally separated by clear spaces (edema), and infiltrated by lymphocytes, plasma cells, and macrophages containing hemosiderin. The gastric lymph nodes showed diffuse sclerosis with abundant collagen fiber deposition and lymphoid tissue atrophy (Fig. 2d-f). No evidence of neoplastic cells or emboli were identified within the lymph nodes. Masson’s Trichrome staining confirmed the diffuse sclerosis in the gastric lymph node.

**Fig. 3:**
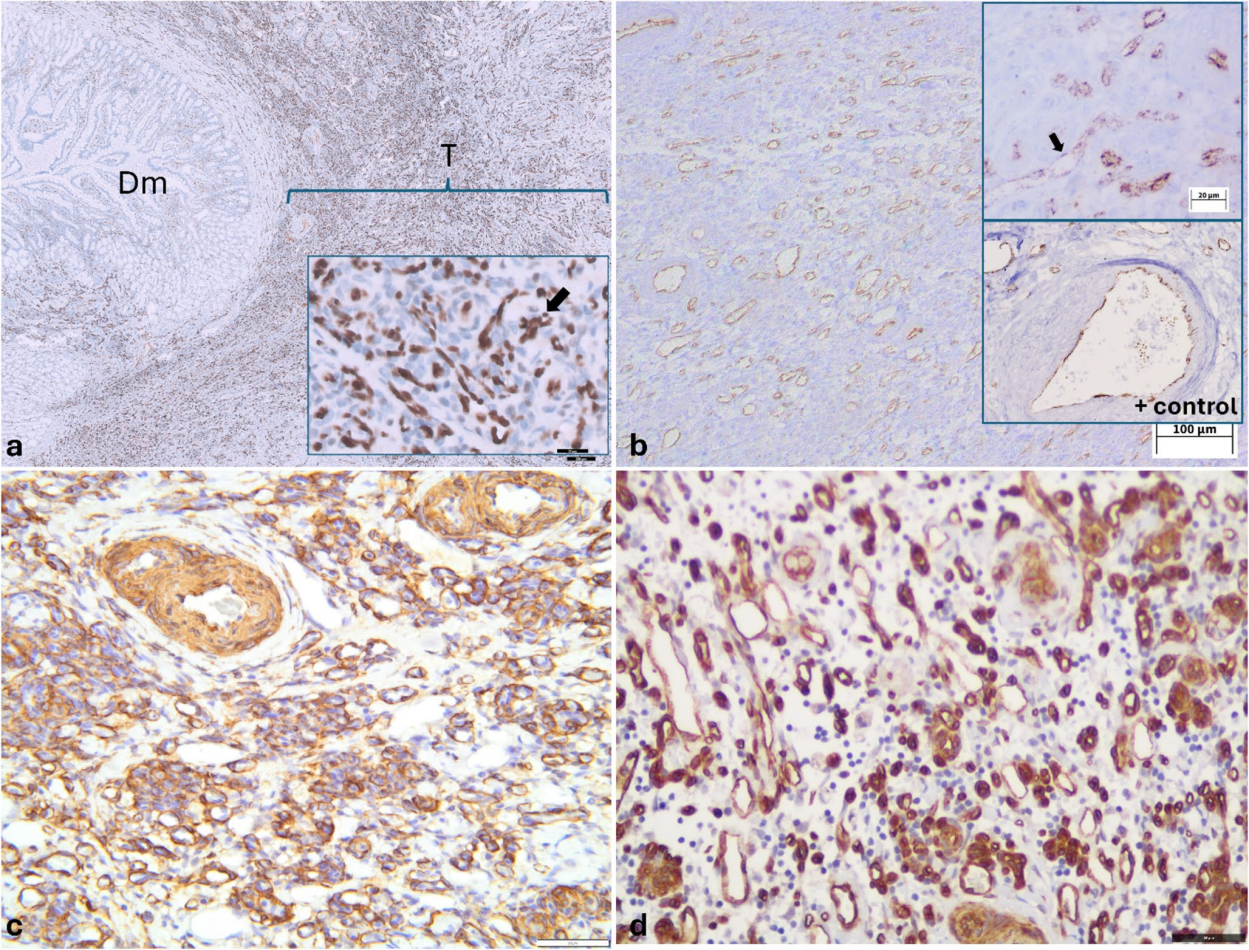
Immunoexpression of ERG, vWF, Cav-1 and α-SMA in the feline gastroduodenal junction KHE (**a**) There is strong immunolabeling for ERG in the nuclei (arrow) of neoplastic endothelial cells; the inset show higher magnification of positive neoplastic endothelial cells (arrow). **b)** Moderate immunolabeling of neoplastic cells for von Willebrand Factor (arrow); the insets show higher magnification of positive neoplastic endothelial cells and positive control. **c)** The endothelial cells are surrounded by α-SMA positive cells. **d)** Strong and diffuse cytoplasmic and membranous Caveolin-1 immunolabeling of neoplastic endothelial cells invading tunica muscularis. Dm (duodenal mucosa), T (tumor). IHC stain

Immunohistochemically, the neoplastic cells showed strong, diffuse nuclear labeling for ERG (Fig. 3a) and patchy, moderate cytoplasmic immunolabeling for vWF (Fig. 3b), further supporting endothelial differentiation. Additionally, α–SMA revealed intense cytoplasmic staining of perivascular cells (Fig. 3c), consistent with a pericytic or muscular phenotype. The neoplastic cells also exhibited cytoplasmic and membranous labeling for Cav-1 (Fig. 3d), supporting endothelial immunophenotype. In contrast, CD31 antibody failed to demonstrate immunoreactivity in either the neoplastic tissue or the control samples.

Based on histological and immunohistochemical findings, a diagnosis of Kaposiform hemangioendothelioma was made.

One-year post-surgery, the patient remained clinically stable with no evidence of disease progression. Follow-up included clinical monitoring and abdominal ultrasonography, which showed no locoregional recurrence, abdominal lymphadenopathy, or detectable metastasis.

## Discussion and conclusion

This report describes the clinical, pathological, and immunohistochemical findings of a case of Kaposiform hemangioendothelioma in a cat, diagnosed at the gastroduodenal junction. The incidence of gastric tumors in cats has increased over the past decades, from 1% in 1970 (Engle and Brodey 1969) to approximately 7% in 2015 (Graf et al. 2015), primarily affecting older individuals, with a mean age of 10–12 years (Negoescu et al. 2025). In this location, lymphoma is the most diagnosed tumor (Paulin et al. 2018), followed by adenocarcinomas and adenomas. Less frequent gastric tumors include leiomyomas, leiomyosarcomas, fibrosarcomas, and gastrointestinal stromal tumors (Negoescu et al. 2025).

Primary vascular proliferations of the digestive system are exceedingly rare, with only isolated cases reported in the literature (Johannes et al. 2007; Toma et al. 2022; Muscatello et al. 2024; Negoescu et al. 2024). To our knowledge, this is the first reported case of gastroduodenal kaposiform hemangioendothelioma in veterinary medicine. Currently, a comprehensive histological classification of vascular neoplasms and malformations in veterinary medicine is lacking, largely due to their rarity and the limited number of reported cases. This underscores the need for further research and standardized diagnostic criteria to improve understanding and management of these complex vascular lesions in animals.

Kaposiform hemangioendothelioma is a rare, locally aggressive vascular neoplasm of borderline malignancy, most commonly diagnosed in infancy or early childhood (Ji et al. 2020). Spontaneous cases have been reported in several animal species, including dogs (posterior limb and right atrium) (Vincek et al. 2004; Paździor-Czapula et al. 2015), a Fischer’s lovebird (neck) (Rossi et al. 2016), and ruminants (urinary bladder and congenital cutaneous lesions) (Pires et al. 2009; Epikmen et al. 2020). Despite varied anatomical locations and clinical presentations, these cases share characteristic histological features, including spindle-shaped endothelial cells, slit-like vascular spaces, and capillary hemangioma–like areas.

The most common clinical signs associated with gastric neoplasia in cats include chronic vomiting, weight loss, anorexia, and lethargy, while less frequent signs include hematemesis, anemia, diarrhea, and a palpable abdominal mass on physical examination (Meuten 2017; Paulin et al. 2018). In human medicine, the clinical manifestation of KHE varies with tumor size and location, ranging from cutaneous lesions or palpable masses to life-threatening complications such as Kasabach-Merritt phenomenon (KMP), a consumptive coagulopathy characterized by severe thrombocytopenia and microangiopathic hemolytic anemia (Ji et al. 2020). In a reported case of intestinal KHE, the clinical signs included progressive abdominal pain, nausea, vomiting, fever, and obstipation, with physical examination revealing a distended, painful abdomen and borborygmi (Aguirre et al. 2019). Similarly, our patient presented with acute vomiting, marked abdominal distention, and severe abdominal pain, consistent with a gastrointestinal disorder, although no evidence of systemic coagulopathy was observed.

Grossly, the tumor may present as a mass or diffuse infiltration of internal organs, potentially resulting in organ dysfunction or compression of adjacent structures (Lyons et al. 2004). In the present case, gastric dysfunction was documented by ancillary investigations, including ultrasonography and gastroduodenoscopy. Intraoperatively, a markedly dense, fibrotic, and hypertrophic pyloric lesion with extensive hyperemia and numerous adhesions was identified, confirming significant impairment of gastrointestinal function. Microscopically, Kaposiform hemangioendothelioma typically appears as a mass exhibiting regions that combine features of both capillary hemangiomas and the vascular slits characteristic of Kaposi’s sarcoma (Ji et al. 2020). The primary histological hallmark of the tumor is characterized by the presence of infiltrating, well-defined, rounded, and confluent nodules. These nodules are composed of spindle endothelial cells featuring flattened nuclei, with minimal atypia and occasional mitoses, which collectively form elongated or curvilinear vascular spaces reminiscent of Kaposi’s sarcoma. Some nodules may contain vessels with round or oval lumens resembling those of capillary hemangiomas, resulting in an intermediate morphological pattern. A characteristic feature, particularly in cutaneous and soft tissue lesions, is the presence of glomeruloid nests composed of rounded or epithelioid endothelial cells (Chundriger et al. 2021). In the present case, similar histological features were observed, including proliferation of vascular endothelial cells forming slit-like vascular spaces, multinodular infiltrative growth, and prominent glomeruloid structures characterized by concentric arrangements of cells around variably sized vascular spaces. These findings are consistent with descriptions of KHE in other animal species, which report nodular infiltrative patterns, spindle-shaped neoplastic endothelial cells, and irregular slit-like vascular spaces interspersed among preexisting collagen bundles. However, in contrast to several veterinary cases (Pires et al. 2009; Paździor-Czapula et al. 2015; Rossi et al. 2016; Vincek et al. 2004), our case showed a more pronounced glomeruloid pattern without areas resembling capillary hemangioma.

The lymph node sclerosis observed in this case is most likely multifactorial and may reflect chronic inflammatory changes, lymphatic congestion, or local microenvironmental remodeling. Given the infiltrative nature of the lesion, secondary lymphatic stasis and sustained inflammatory signaling could plausibly promote fibroblast activation and collagen deposition within the draining lymph node. In addition, tumor-associated cytokines and growth factors may contribute to extracellular matrix accumulation and sclerosis (Saxena et al. 2019; Bois et al. 2021). Therefore, these changes should be interpreted as associated findings rather than evidence of a direct causal relationship. Differential diagnosis is essential due to the variable biological behavior of these tumors. Kaposi hemangiosarcoma’s characteristic marked cellular atypia and high mitotic index along the presence of PAS-positive hyaline globules, were not observed in the current case, distinguishing it from Kaposiform hemangioendothelioma (Hasby et al. 2016; Roccabianca et al. 2020). The main differential diagnoses, based on macroscopic, histological, and immunohistochemical features, along with their prognostic implications, are summarized in Table 1.

**Table 1:** Comparative features of the principal differential diagnoses of vascular lesions

Lesion	Gross features	Histology and immunohistochemistry	Prognosis
Kaposi’s sarcoma/ hemangiosarcoma (Roccabianca et al. 2020)	Often poorly circumscribed; may show necrosis, hemorrhage, and areas of tissue destruction.	Marked cellular atypia, high mitotic index, necrosis, hemorrhage, and absence of cannonball morphology The immunohistochemical panel for endothelial markers (CD31, CD34, FVIII, LYVE-1), LNA-1/HHV-8 for the presence of Herpesvirus (Pantanowitz et al. 2010).	Poor: hemangiosarcoma are highly infiltrative masses with high metastatic potential.
Capillary hemangioma (Dilsiz et al. 2009; Kim et al. 2023)	Small, spherical to polypoid, well-circumscribed nodules, soft to firm in consistency, dark red to black in color, with a spongy appearance.	Composed of lobules of capillaries lined by a single layer of flat to plump endothelial cells; mitotic figures are rare to absent. It displays a benign endothelial phenotype (CD31+, CD34+, vWF+), surrounded by SMA+ pericytes and weaker or absent expression in developing mural layer, compared with normal vessels.	Excellent, with complete surgical excision.
Spindle cell hemangioma	Solitary nodules with smaller satellite lesions in the same area (Suzuki et al. 2018).	More circumscribed, not multilobulated, lacks an area with a glomeruloid pattern, biphasic structure: cavernous/vascular spaces and solid cellular areas. The immunohistochemical panel for endothelial markers (CD31, CD34, FVII, ERG), SMA+ spindle cells arranged in solid areas (Tosios et al. 2008; Roccabianca et al. 2020; Hu et al. 2022).	Excellent: no metastatic potential, no malignant transformation in untreated lesions or recurrence (Perkins and Weiss 1996; Yamazaki et al. 2023).
Epithelioid hemangioendothelioma	Well-demarcated, red or dark-red nodules (Muscatello et al. 2024)	Epithelioid endothelial cells with intracytoplasmic vacuoles occasionally contain erythrocytes. Immunohistochemically, the cells are CD31+, FVIII+, CD34+ (Yaman et al. 2018; Muscatello et al. 2024).	Guarded, due to indolent-to-progressive behavior, rare metastatic (Kou et al. 2020).
Feline systemic reactive angiomatosis (Fuji et al. 2005; Yamamoto et al. 2015; Espenica et al. 2025).	Multiple organs are involved; the changes depend on the degree of vascular occlusion: thickening, dark red, firm areas, mild congestion, fibrosis or infarcts, nodules.	Occlusive, multifocal, intraluminal proliferations of endothelial cells and pericytes within small vessels; the lesion comprises a dominant endothelial population (vWF+, CD31 + and a smaller pericytic/mural component (SMA±, desmin±), supporting a dual endothelial–pericytic composition.	Poor, due to rapid progress and no successful treatment.

In addition to histological findings, immunohistochemistry using specific antibodies for endothelial and perivascular cells is required. Veterinary studies on vascular neoplasms have primarily focused on traditional endothelial markers such as CD31 and von Willebrand Factor (Gamlem and Nordstoga 2008), while the evaluation of ERG and Cav-1 has not been reported in feline cases to date. *In silico* homology analyses demonstrated a high degree of amino acid sequence conservation between human and feline ERG and caveolin-1, particularly within the regions corresponding to antibody epitopes, supporting the likelihood of cross-reactivity of the antibodies used in this study. ERG showed especially strong conservation within the C-terminal epitope region, while caveolin-1 exhibited high overall and immunogen-specific sequence identity. Von Willebrand Factor showed a slightly lower degree of amino acid sequence conservation between human and feline sequence, compared with the first ones. In comparison, PECAM-1 (CD31), included as a reference endothelial marker with established veterinary use, displayed lower overall homology between human and feline sequences. In both human and animals, CD31, a well-characterized transmembrane glycoprotein expressed on endothelial cells, platelets, and leukocytes, is used for confirming endothelial differentiation in various neoplasms (Jennings et al. 2012). However, immunohistochemical application in animals can be limited by antibody specificity and tissue compatibility (Webster et al. 2020). In this study, the available CD31 antibody clones did not produce reliable staining in feline tissue, underscoring the need to explore additional endothelial markers to improve tumor characterization.

The Erythroblastosis Transformation-Specific Regulated Gene 1, a highly specific marker for endothelial differentiation, plays a crucial role in angiogenesis and homeostasis (Birdsey et al. 2011). In human medicine, studies demonstrated superior diagnostic performance for ERG compared with CD31, with 100% sensitivity in hepatic angiosarcoma and consistent expression across other vascular tumor types (Wang 2014; Haber et al. 2015; Sullivan et al. 2015). In the present case, the neoplastic cells showed diffuse and intense positivity, confirming their endothelial origin. These findings suggest that this marker could serve as a potential alternative tool for the immunohistochemical diagnosis of vascular neoplasms in animals; however, further studies involving larger case series are required.

Currently, Cav-1 is being investigated as a new potential therapeutic target due to its role in angiogenesis, cell signaling, and tumor progression. A study conducted by Morgan et al. (2001), found significant differences in the expression of this antibody in dermal vascular tumors, with benign tumors showing high caveolin-1 expression, while malignant neoplasms presented markedly reduced expression. This protein is involved in tumor invasion and metastatic behavior by several mechanisms, including invadopodia formation, interfering with cytoskeletal dynamics and metalloproteinase activity (Senetta et al. 2013). In vascular tumors, a decreased expression can be associated with immature, unstable vasculature, decreased pericyte coverage and a more aggressive biological behavior (DeWever et al. 2007). Resembling human studies, in our case the pattern and intensity of Cav-1 expression in the neoplastic endothelial cells was similar to those found in normal blood capillaries from the mucosal lamina propria, suggesting a well differentiated neoplastic tissue.

Accordingly, the tumor’s immunoreactivity for ERG and Cav-1 in this case confirms the vascular origin of the neoplastic cells. Although research on these markers in veterinary vascular neoplasms is limited, their established significance in human vascular tumors indicates substantial diagnostic and prognostic potential.

Kaposiform hemangioendothelioma cells show positivity for both endothelial markers and smooth muscle actin (SMA), particularly in perivascular regions, reflecting vascular tumors with pericyte or smooth muscle differentiation (Chundriger et al. 2021), and these findings are in line with our results. KHE exhibits intermediate biological behavior, with locally infiltrative and aggressive growth but low metastatic potential (Lyons et al. 2004). In veterinary medicine, the limited number of cases restricts understanding of long-term behavior and prognosis, with only one case of canine cutaneous KHE showing no metastasis 6 months post-surgery (Vincek et al. 2004). Following complete surgical excision in our patient, remarkable recovery was observed with no recurrence or metastasis after one year.

Kaposiform hemangioendothelioma is a rare vascular neoplasm, particularly in non-human species, and this represents the first documented case involving the alimentary tract in animals. Here, we report the clinicopathological features, immunohistochemical profile, and one-year treatment outcome of a feline gastroduodenal junction KHE. We also showed that ERG and Cav-1 may serve as a potential alternative tool for the immunohistochemical diagnosis of vascular neoplasms in cats. Vascular tumors should be considered in the differential diagnosis of gastrointestinal masses in cats, given their potential for locally aggressive behavior and associated complications.

## Data Availability

The original contributions presented in the study are included in the article/supplementary material, further inquiries can be directed to the corresponding author.

## Declarations

This research was supported by the University of Agricultural Sciences and Veterinary Medicine Cluj-Napoca.
